# Hospitalisation rates for youth living with perinatally acquired HIV in England

**DOI:** 10.1371/journal.pone.0295639

**Published:** 2024-03-19

**Authors:** Sarah May Johnson, Jhia Jiat Teh, Thomas Joshua Pasvol, Sara Ayres, Hermione Lyall, Sarah Fidler, Caroline Foster

**Affiliations:** 1 Department of Paediatric Infectious Diseases, Imperial College Healthcare NHS Trust, London, United Kingdom; 2 Department of Infectious Diseases, Imperial College London, London, United Kingdom; 3 Department of HIV Medicine, Imperial College Healthcare NHS Trust, London, United Kingdom; 4 University College London, London, United Kingdom; Emory University School of Medicine, UNITED STATES

## Abstract

**Introduction:**

Complex challenges amongst ageing cohorts of adolescents and adults living with perinatally acquired HIV (PaHIV) may impact on hospitalisation. We report hospitalisation rates and explored predictive factors for hospitalisation in adolescents and adults (10–35 years) living with PaHIV in England.

**Method:**

Retrospective observational cohort study over a three-year period 2016–2019. Data collected included cause and duration of hospitalisation, HIV viral load and CD4 lymphocyte count. The primary outcome was overnight hospitalisation. Patients exited at study end/ transfer of care (TOC)/ loss to follow up (LTFU) or death. Maternity/hospital admissions at other centres were excluded. Admission rates per 100 person-years (95% CI) were calculated by age group. Negative binomial regression with generalized estimating equations was performed.

**Results:**

255 patients contributed 689 person-years of follow up. 56% were female and 83% were of a Black, Black British, Caribbean or African ethnicity. At baseline, the median age was 19 years (IQR 16–22). 36 individuals experienced a total of 62 admissions which resulted in 558 overnight stays (median stay was 5 nights). One person died (lymphoma), six had TOC and one was LTFU by the end of the three-year study period. Crude incidence of admission for the whole cohort was 9.0 per 100 PY (6.9–11.6). The respective crude incidence rates were 1.5 PY (0.0–8.2) in those aged 10–14 years and 3.5 PY (1.5–7.0) in the 15–19-year-olds. In those aged 20–24 years it was 14.5 PY (10.1–20.2) and in those >25 years the crude incidence rate was 11.7 PY (6.9–18.5). Factors significantly associated with admission were a CD4 lymphocyte count <200 cells/uL, adjusted IRR 4.0 (1.8–8.8) and a history of a CDC-C diagnosis, adjusted IRR 2.9 (1.6–5.3). 89% admissions were HIV-related: 45% new/current CDC-C diagnoses, 76% due to infection.

**Conclusions:**

Hospitalisation rates were four-fold higher in adults (>20 years of age) compared to adolescents (10–19-year-olds). The continuing challenges experienced by PaHIV youth require enhanced multidisciplinary support throughout adulthood.

## Introduction

An estimated five million young people aged 15–25 years live with HIV (human immunodeficiency virus) infection worldwide [1]. Preventing hospitalisation is a key element of the HIV response. Since the late 1990s there have been declining rates of hospitalisation amongst both adults and children living with HIV attributable to the advent and advances of antiretroviral therapy (ART), improved case identification and test and treat strategies [2–4].

In the UK, annual hospital admission rates fell from 69 per 100 children living with PaHIV in 2000 to 5 per 100 in 2018 [5]. In adults living with HIV, despite a reduction in hospitalisation [3], rates remain higher than the general population [6, 7]. For example, in the UK, adults living with behaviourally acquired HIV have admission rates of 2.7 per 100 person years after their first year of diagnosis [8, 9].

Adolescents and young adults who are living with perinatally acquired HIV (PaHIV) are the only groups in which HIV-associated mortality and morbidity continues to rise; attributed to the long-term survivors of the perinatal epidemic [10]. Living with PaHIV involves infection during early neurodevelopment, complex ART regimens [11] and the impact of chronic infection and inflammation [12]. These factors may continue to impact on quality of life, including hospitalisation, beyond childhood and adolescence. Emerging data suggests the main predictor of morbidity and mortality from early adulthood and beyond is poor disease control in childhood including history of prior CDC-C (US Centers for Disease Control and Prevention Category-C diagnoses), CD4 count and viraemia on leaving paediatric care [9, 13–16].

Hospitalisation of youth living with PaHIV throughout adolescence and into adult care is poorly characterised [17] with research largely focused on 0–14 and 15+-49 years of age, the latter infrequently disaggregated by age [12, 18, 19]. Published data from 2014 reported hospitalisation rates of 17.1 per 100 PY for children and young adults living with PaHIV the US, although more recent data, including from the European region remains sparse [20].

Our primary aim was to explore causes and rates of hospitalisation in adolescents and adults living with PaHIV over a three-year period in a single regional centre in England. Characterising hospitalisation highlights the illnesses affecting this cohort and insight into the lived experience of young adults, in whom hospitalisation will involve disruption to education and work. It is hypothesised that factors associated with admission would include those with poor disease control (i.e. a low CD4 cell count and/or high viral load). We further aimed to describe the cause and duration of admissions and to compare the admission rates by age grouping.

## Materials and methods

We conducted a retrospective observational cohort study including all PaHIV individuals aged >10 years attending a specialist centre in London between 1st September 2016-31st August 2019. All care including ART is provided free. This specialist centre provides care for both paediatric and adult patients. Patients have telephone access to clinicians by text, and mobile and direct open access to clinic whereby they can walk into a once weekly clinic without a pre-arranged appointment. If required patients can be admitted directly from clinic. This cohort can receive care from both paediatric and adult HIV services through the same clinic space but with different clinical teams. The median age of transition to adult outpatient HIV services was 17.8 years (range 15.2–20.1). The service has been described in detail previously [10, 21].

Patients exited the study at the earliest timepoint of date study ended/ transfer of care (TOC) (defined as documented evidence of transfer of care)/ loss to follow up (LTFU) (defined as no clinical contact for >1 year) or death. The primary outcome was any and all overnight hospitalisations. The primary diagnosis for each admission was collected and characterised. The notes and diagnosis were reviewed by two senior clinicians and categorised as infective or non-infective. A second approach was explored by categorising admissions as AIDS related with a CDC-C diagnosis, HIV related but not a CDC-C diagnosis and non-HIV related e.g. surgery. Maternity admissions were excluded as they were likely to increase with age. All admissions to other healthcare facilities were excluded due to incomplete data.

Demographic and clinical data were collected with manual abstraction from the electronic patient records. Sex was defined as biological sex at birth. Ethnicity was categorised as Black, Black British, Caribbean or African and other ethnicities which included: White, Mixed, Asian and other ethnicities. Age groups were selected based on WHO (World Health Organisation) definition of adolescence 10–19 (‘young adolescence’ as 10–14 years) [22] and 20–24 year olds as young adults. Patients were categorised into four age groups: younger adolescents 10–14, older adolescents 15–19, young adults 20–24 and adults 25–35 years old. These were assigned according to each patient’s age at the mid-point of each year of follow up (each year defined as: beginning on 1^st^ September for each year- 31^st^ August the following year 2016–2017, 2017–2018 and 2018–2019).

The prior history of a CDC-C diagnosis was a binary yes/no variable (which dated from the neonatal period onwards) and was updated each year, thus if the patient had ‘ever had’ a CDC-C diagnosis they continued to be categorised as such for the whole period of the follow up. Evidence of ever having had any CDC-C diagnosis was captured at the start of each year of follow up. If a patient had their only and new CDC-C diagnosis, for example during the 1^st^ year (2017–2018) they would then be classified as having a history of a CDC-C diagnosis in the 2018–2019 time period. For plasma HIV viral load and CD4 count, the first measurements of each follow up year were used (for the time periods detailed above).

Rates of admission per 100 PY (95% CI) were calculated by age group. Analysis was performed using negative binomial regression with generalized estimating equations. Stata^TM^ 16.1 was used for all analyses.

This project was registered as a service evaluation with the audit office. Ethical approval was not required as per UK Health Research Authority (HRA) guidance for routinely collected clinical data as part of an anonymised database for clinical decision making. A previous wider service evaluation has prior publication [10, 21].

## Results

Two hundred and fifty-five individuals living with PaHIV contributed 689 person-years of follow up. Fifty six percent (n = 143) were female, 83% (n = 212) were of a Black, Black British, Caribbean or African ethnic group (Table 1). The proportion of patients in each age group at the beginning of the follow up period in 2016 are described in Table 1. At the beginning of the follow up period, the median age was 19 years (IQR 16–22), HIV viral load (VL) was <200 copies/ml in 77% (n = 197) of patients and 89% (n = 226) had a CD4 count >200 cells/μl. One person died of a lymphoma during the three-year period with a further 2.7% (n = 7) exiting before the follow up ended; six patients due to TOC and one was LTFU. Thirty two percent had one or more prior CDC-C diagnoses including: pulmonary *Mycobacterium tuberculosis* 14, infantile HIV encephalopathy 13, Cytomegalovirus disease 13, *Pneumocystis jiroveci* pneumonia (PJP) 12, *Non-tuberculosis mycobacterium* 6, lymphoma 4, Kaposi’s sarcoma 2, oesophageal candidiasis 3 and cerebral toxoplasmosis 2.

**Table 1:** Cohort characteristics at the beginning of follow up (2016).

	n (%)/ median (IQR)
**Age (years)**	19 (16–22)
10–14	44 (17%)
15–19	102 (40%)
20–24	74 (29%)
>25	35 (14%)
**Birth sex**	
Female	143 (56%)
Male	112 (44%)
**Ethnicity**	
Black, Black British, Caribbean or African	212 (83%)
Other (White, Mixed, Asian, Other)	43 (17%)
**HIV viral load (copies/ml)**	
Viral suppression (<200)	197 (77%)
Viral load detectable (>200)	58 (23%)
**CD4 count (μl)**	651 (455–841)
<200	30 (12%)
>200	226 (88%)
**Lost to follow up/ transferred care**	7 (2.7%)
**Died during study period**	1 (<0.5%)

### Admissions

Sixty-two admissions occurred amongst 36/255 (14%) individuals, resulting in 558 admission nights (median five nights, IQR 2–9, range 1–103). Thirty percent of the 36 individuals experienced more than one hospitalisation. One patient had seven admissions, another two had four admissions with three experiencing three separate admissions. Eight patients had two admissions and the remaining patients a single admission. Nine admissions occurred before the age of 20 years and 53 admissions (85%) occurred from the ages of 20–35 years.

At the time of admission, 61% (38/62) had a detectable viral load (>200 copies/ml), 66% (41) had a CD4 count <350 cells/μl and 56% (35) a CD4 count <200 cells/μl. Furthermore, 92% (13/14) of those requiring more than one admission were aged 20–35 years, 78% (11) had detectable viraemia and 64% (9) a CD4 count <200 cells/ul at the start of follow up in 2016.

**Fig 1:**
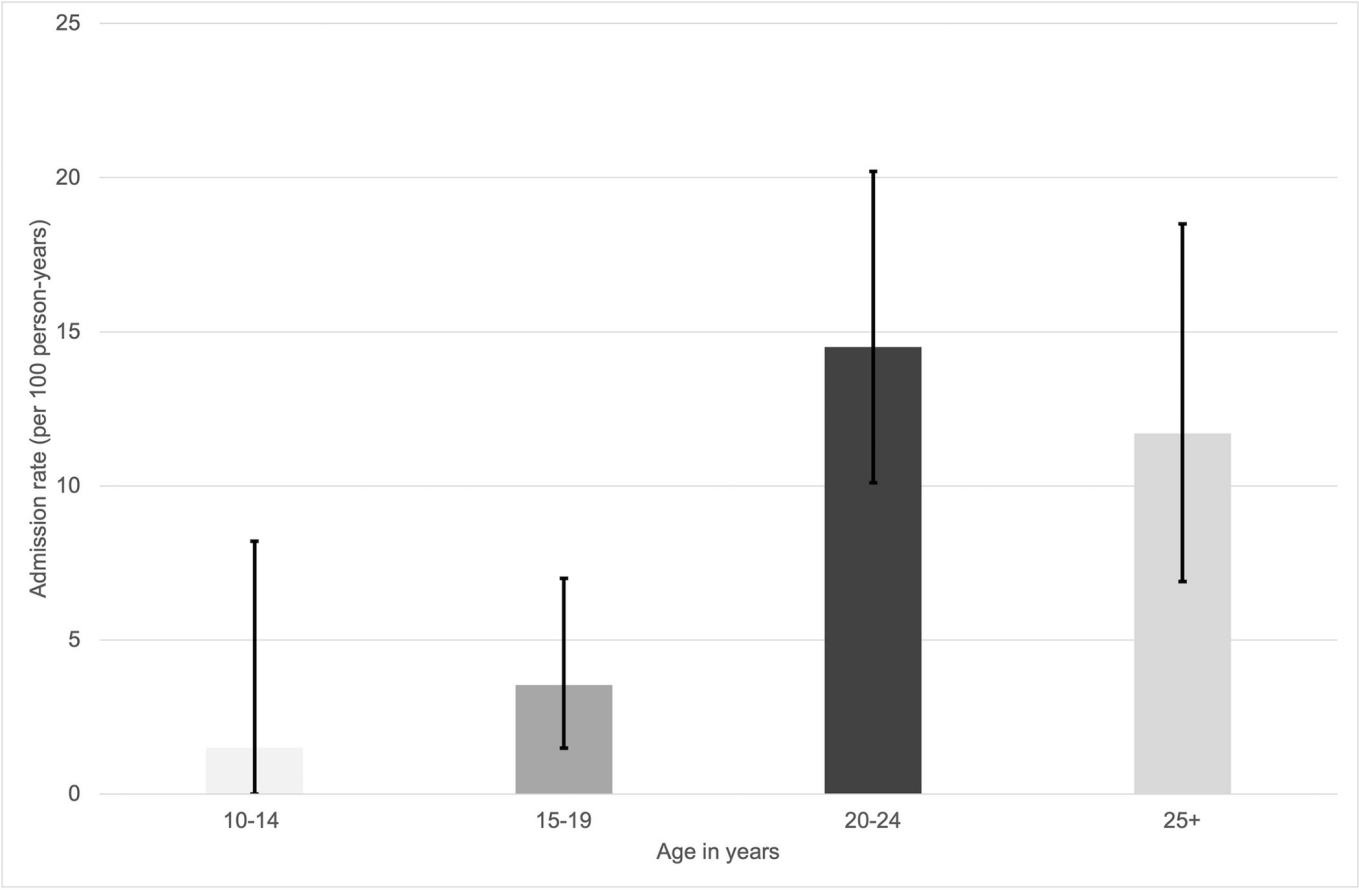
Admission rates (crude incidence rate per 100 person years) for the different age groups.

The overall crude incidence of admission was 9.0 per 100 PY (95% CI, 6.9–11.6). The crude admission rates for respective age groups are show in Fig 1. For the 10–14-year-olds the admission rate was 1.5 per 100 PY (95% CI, 0.0–8.2), the 15–19-year-olds had a rate of 3.5 per 100 PY (95% CI, 1.5–7.0). The admission rate for those who were 20–24 years was 14.5 per 100 PY (95% CI, 10.1–20.2) and for the 25+ year olds it was 11.7 per 100 PY (95% CI, 6.9–18.5).

There was a trend in a reduction in admissions over time from 2016–2017 through 2017–2018 and to 2018–2019, with the least admissions occurring during 2018–2019, although this was not statistically significant (see Table 2). Time varying covariates significantly associated with admission included: a low CD4 count, adjusted IRR 4.0 (1.8–8.8) and a CDC C-diagnosis, adjusted IRR 2.9 (1.6–5.3) (see Table 2).

**Table 2:** Crude incidence rates (IR) and adjusted incidence rate ratios (IRR) of admission for PaHIV adolescents and young people by year, age group, birth sex, ethnicity and characteristics at the start of follow up in 2016: History of CDC-C diagnosis, HIV viral load and CD4 lymphocyte count.

Variable	Number of admissions N (%)	Crude IR (95% CI)	Adjusted* IRR (95% CI)**	*p* value
**Overall**	62	9.0 (6.9–11.6)		
**Period**				
2016–2017	28 (45%)	12.5 (8.3–18.1)	1.3 (0.8–2.0)	0.3
2017–2018	23 (37%)	10.0 (6.3–15.0)	1	
2018–2019	11 (18%)	4.7 (2.3–8.4)	0.5 (0.3–1.0)	0.07
**Age (years)**				
10–14	1 (2%)	1.5 (0.0–8.2)	1	
15–19	8 (13%)	3.5 (1.5–7.0)	1.4 (0.2–12.6)	0.7
20–24	35 (56%)	14.5 (10.1–20.2)	2.3 (0.3–19.2)	0.4
25+	18 (29%)	11.7 (6.9–18.5)	3.1 (0.4–28.1)	0.3
**Birth sex**	** **			
Male	22 (35%)	7.2 (4.5–10.9)	1	
Female	40 (65%)	10.4 (7.5–14.2)	1.96 (0.9–2.5)	0.06
**Ethnicity**				
Black, Black British, Caribbean or African	54 (87%)	9.3 (7.0–12.2)	1.6 (0.8–3.2)	0.2
Other (White, Mixed, Asian)	8 (13%)	7.3 (3.2–14.4)	1	
**Previous history of CDC-C diagnosis**	** **			
Yes	48 (77%)	19.8 (14.6–26.4)	2.9 (1.6–5.3)	0.001
No	14 (23%)	3.3 (1.9–5.5)	1	
**HIV viral suppression (<200 copies/ml)**				
Yes	15 (24%)	3.5 (2.1–5.5)	1	
No	47 (76%)	29.4 (21.3–39.7)	1.7 (0.8–3.8)	0.2
**CD4 count <200 μl**				
Yes	37 (60%)	51.8 (37.0–70.5)	4.0 (1.8–8.8)	0.001
No	25 (40%)	3.6 (2.2–5.5)		

*Adjusted for other variables considered; year, age group, birth sex, ethnicity, HIV VL, history of CDC-C diagnosis, CD4 lymphocyte count, respectively

**Adjusted IRRs compared to the reference group for each categorical variable

Eighty nine percent (55 admissions) of the total of 62 admissions were HIV-related, of which new and current CDC-C diagnoses accounted for 45% (28) of total admissions. Of these, the commonest were Mycobacterial infections 11% (7), HIV wasting 8% (5), presumed/suspected PJP 6% (4), Herpes Simplex Virus infections (HSV) 6% (4), candidiasis 5% (3) and lymphoma 5% (3), Other HIV-related (non-CDC-C) causes accounted for a further 45% (27) of admissions. These included symptomatic HIV (off ART), respiratory tract infections including influenza, hepatic and cardiac non-cirrhotic portal hypertension secondary to HIV, efavirenz related drug reaction, corneal graft secondary to HIV keratoconus.

Hospitalisations were also categorised as infective or non-infective. Seventy six percent (47) of the 62 admissions were due to one or more infections; respiratory infections predominated 37% (23), with gastrointestinal 19% (12), dermatological 6% (4), symptomatic HIV after stopping ART 3% (2). CNS infections occurred in 3 (5%) (1 viral meningitis, 1 bacterial meningitis, 1 cerebral toxoplasmosis) with single cases of disseminated HSV, sepsis and urinary tract infections. Non-infective causes included surgery 5% (3; corneal graft, orchidectomy, bilateral ovarian cystectomy), psychoses 5% (3), asthma 3% (2), non-cirrhotic portal hypertension 3% (2) and efavirenz related drug reaction 2% (1).

## Discussion

Within our service, hospitalisation rates in young adults (20–24 year olds) living with perinatally acquired HIV were four times higher than in late adolescence (15–19 year olds). Our finding that those older than 25 years of age, had the second highest incidence of admission may highlight the impact of the pre-ART era on hospitalisation later in life. ART was established in the UK from 1996, therefore individuals aged 20 and over at study entry would have experienced early life without ART, viraemia and HIV related morbidity in infancy which may continue to impact into adulthood [10, 23, 24].

Our findings suggest that adulthood, not adolescence is the time period when our cohort of patients living with PaHIV are most likely to be admitted to hospital. A similar but more modest trend is seen in other chronic conditions of childhood e.g. type 1 diabetes [25]. Emerging UK data suggests that the main driver for poor outcomes in adult HIV care is exiting paediatric care with markers of disease progression including unsuppressed viraemia, a lower CD4 count and a previous CDC-C diagnosis [9, 13, 16]. Our findings identified that risk factors for hospitalisation included a previously low CD4 count and a prior CDC C-diagnosis. These risk factors can assist clinicians by highlighting which patients might benefit from more intensive and enhanced care throughout young adulthood.

Despite viral suppression rates approaching 80% in our cohort, one in five continue to struggle with adherence to ART. Indeed, the majority (89%) of hospital admissions in our cohort are HIV-related. This is in stark contrast to admissions of adults living with behaviourally acquired HIV in well-resourced settings where non-AIDS related causes predominate [8, 26, 27].

Infection was the most common cause of admission with respiratory infections predominating in our study. This is similar to children and adults in resource-limited settings not on suppressive ART where infections are among the commonest causes of admission [27]. Our findings are in contrast to hospitalisation of adults living with behaviourally acquired HIV in London, in who non-infectious causes accounted for most admissions (circulatory and digestive diseases were the most common causes) [26].

One of the strengths of our study is the manual extraction and review of all electronic notes with review from the senior clinician of each patient to define primary cause for admission, as opposed to reliance solely on electronic databases codes [20]. However, the patients admitted comprise a relatively small sample size which should be taken into account when considering our results. Whilst this study captures the majority of hospital admissions in this cohort, it will be an underestimation because hospital admissions elsewhere and mental health admissions to psychiatric wards were not included. Paediatric HIV admissions (<18 years) are almost always to the tertiary centre, whilst admissions in adult care may include local hospitals, particularly for those in full time tertiary education (at university/college across the UK). As such, the underestimation is likely to be greater for those aged 20–35 than 10–19-year-olds. Other limitations include that those over 25 years were born in the pre-ART era and hence represent a specific group of slow progressors of people living with PaHIV. Our findings are useful to clinicians managing adults living with PaHIV, especially those born in the pre-ART era. However, due to the changing picture of health in the post-ART era, our study may lack generalisability to future and younger cohorts.

In conclusion, we found that adults living with PaHIV have markedly higher rates of hospitalisations than adolescents. The majority of these admissions were HIV-related with infections causing most hospital admissions. Our findings highlight the continuing adherence challenges for adults born with HIV and the need for enhanced multidisciplinary support that starts in paediatric care and continues through the life course.
